# Multi-omics comparison of two emerging storage pests (*Necrobia rufipes* and *Tribolium castaneum*) of dried black soldier fly larvae product

**DOI:** 10.1038/s41598-025-34902-7

**Published:** 2026-01-05

**Authors:** Inusa Jacob Ajene, Chrysantus M. Tanga, Komivi S. Akutse, Samantha W. Karanu, Fathiya M. Khamis

**Affiliations:** 1 International Centre of Insect Physiology and Ecology, Nairobi, Kenya; 2 College of Tropical Agriculture and Human Resilience, Komohana Research and Extension Center, University of Hawaii at Manoa, Hilo, Hawai’i 96720 USA; 3 Unit for Environmental Sciences and Management, North-West University, Potchefstroom, 2520 South Africa; 4 Department of Zoology and Entomology, University of Pretoria, Hatfield, South Africa

**Keywords:** Gut microbiome, Mitogenome, Postharvest storage pest, Red flour beetle, Red-legged ham beetle, Ecology, Ecology, Microbiology, Zoology

## Abstract

The black soldier fly (BSF) larvae is a rich and promising source of alternative protein that continues to increasingly gain global traction as a functional ingredient for sustainable livestock and fish production. The key setback to postharvest processing of stored BSF larvae (BSFL) products is the significant damage caused by two notable storage pests (*Tribolium castaneum* and *Necrobia rufipes*). Here, we present a comparative analysis of the complete mitochondrial genomes and gut microbiome profiles of *T. castaneum* and *N. rufipes*. The study mitogenomes were similar in size and structure to other coleopteran mitogenomes. The gut microbiome profiles of the two pests showed a high abundance of bacteria in the Proteobacteria and Firmicutes phyla. However, *T. castaneum* had 78% more phyla represented within its microbiome than *N. rufipes*. The most abundant genera in *T. castaneum* were *Staphylococcus* and *Streptococcus*, while in *N. rufipes*, the dominant genera were *Klebsiella* and *Synechococcus*. We also identified the presence of potentially clinically harmful microbial genera (*Stenotrophomonas maltophilia*) in the gut of *T. castaneum* and *N. rufipes* in relatively high abundance. These results provide insight into potential harmful associations in the gut of the storage pest, picked from contaminated, poorly processed BSFL products.

## Introduction

The use of insect-based meal as an alternative and sustainable protein source for humans and livestock has rapidly gained global attention in recent years^1^. The increasing global population and consequent increase in the demand for food and feed have accelerated the need for alternative protein sources, particularly for animals^2^. Among these insects, the black soldier fly (BSF), *Hermetia illucens* L. (Diptera: Stratiomyidae), has been shown to have immense potential as a promising ingredient for animal feed and other circular-economy applications. The BSF is efficient in recycling organic waste into high-quality, valuable products (protein, oils, frass fertilizer, chitin/chitosan, and other bioactive compounds)^3^. The BSF larvae have been used to replace conventional protein sources in livestock feed such as poultry, swine, and fish^4–8^. Long-term storage of BSF larvae products, as with other highly nutritious grains, is prone to attack by pests and pathogens in storage^9,10^. Two key pests of stored BSF larvae products that have been identified are the flour beetle, *Tribolium castaneum* Herbst, 1797, and the red-legged ham beetle, *Necrobia rufipes* De Geer 1775^11^. *Tribolium castaneum* is a widespread generalist pest on stored food products worldwide and typically completes its lifecycle in flour or other dried foods^12,13^. However, the recent identification of this pest on stored BSF larvae reflects host expansion of the pest from carbohydrate-rich, grain-based substrates such as maize (> 70% carbohydrates) to protein- and fat-rich substrates such as BSF larvae^14^. This warrants a critical evaluation of the pest’s biology, as *T. castaneum* has been shown to be a model insect in research and a preferred candidate for developmental studies and genomic comparisons among insect species^15^.

On the other hand, *N. rufipes* has been identified as an important economic pest species infesting dried or smoked fish, meat, and insect products in storage^16^. Furthermore, it is also the most injurious member of the Cleridae family, particularly in commodities that are susceptible to infestation by stored product pests in retail stores^17^. It is generally reported in stored products which are rich in protein content, and recently it has been reported on stored BSF larvae^18,19^. The recent identification of the two pests (*N. rufipes* and *T. castaneum*) on stored BSF larvae poses a serious problem in the BSF value chain, with the potential for significant economic losses (qualitative and quantitative) to farmers and producers^20–22^. Furthermore, the potential proliferation and/or transmission of pathogenic microbes belonging to the genera *Stenotrophomonas*, *Clostridia*, *Staphylococcus*,* Campylobacter*, and *Streptococcus*, which could be harboured within the gut or on the exoskeleton of these pests, is a cause for concern^20–22^. However, information on the biology of this pest in relation to its genetic disposition to host selection and microbiome composition is lacking. Therefore, studies into the biology of the pests are necessary to identify sustainable mitigation strategies. The study of insect mitochondrial genomes has provided deep insights into species phylogeny due to the highly degenerate circular genome and the cladistically informative gene order arrangements^23^. Also, the mitochondrion facilitates key metabolic pathways, including the tricarboxylic acid (TCA) cycle, which is involved in protein, lipid and carbohydrate metabolism^24^. This could provide valuable information on the genetic basis for host selection and host range expansion in these pests. Numerous Coleopteran mitogenomes have been studied, including *T. castaneum*^25–28^. However, mitogenomes of major groups, including *N. rufipes*, are still missing. Furthermore, the microbiome communities of these two pests found on stored BSF larvae need to be elucidated as the microbiomes of most insects have been shown to modulate different aspects of their biology, including reproduction, development, and behaviour^13,29,30^. The co-occurrence of these two pests on stored BSF larvae warrants a comparative look into their microbiome compositions to provide a biological basis for developing sustainable preventative measures against the pests. In line with this background, our study sought to provide knowledge on the mitochondrial genomics and microbiome composition of these two emerging storage pests of dried BSF larvae product in comparative analyses and identify interplays between the genetic diversity of the species and their microbiome community diversity. In addition to the complete mitochondrial genome of *T. castaneum*, we hereby present the complete mitochondrial genome sequence of *N. rufipes* to extend the taxonomic range of the family Cleridae, aid species identification, and support phylogenetic analysis for placement with closely related species.

## Results

### Mitogenome features and comparison between *Necrobia rufipes* and *Tribolium castaneum*

**Fig. 1:**
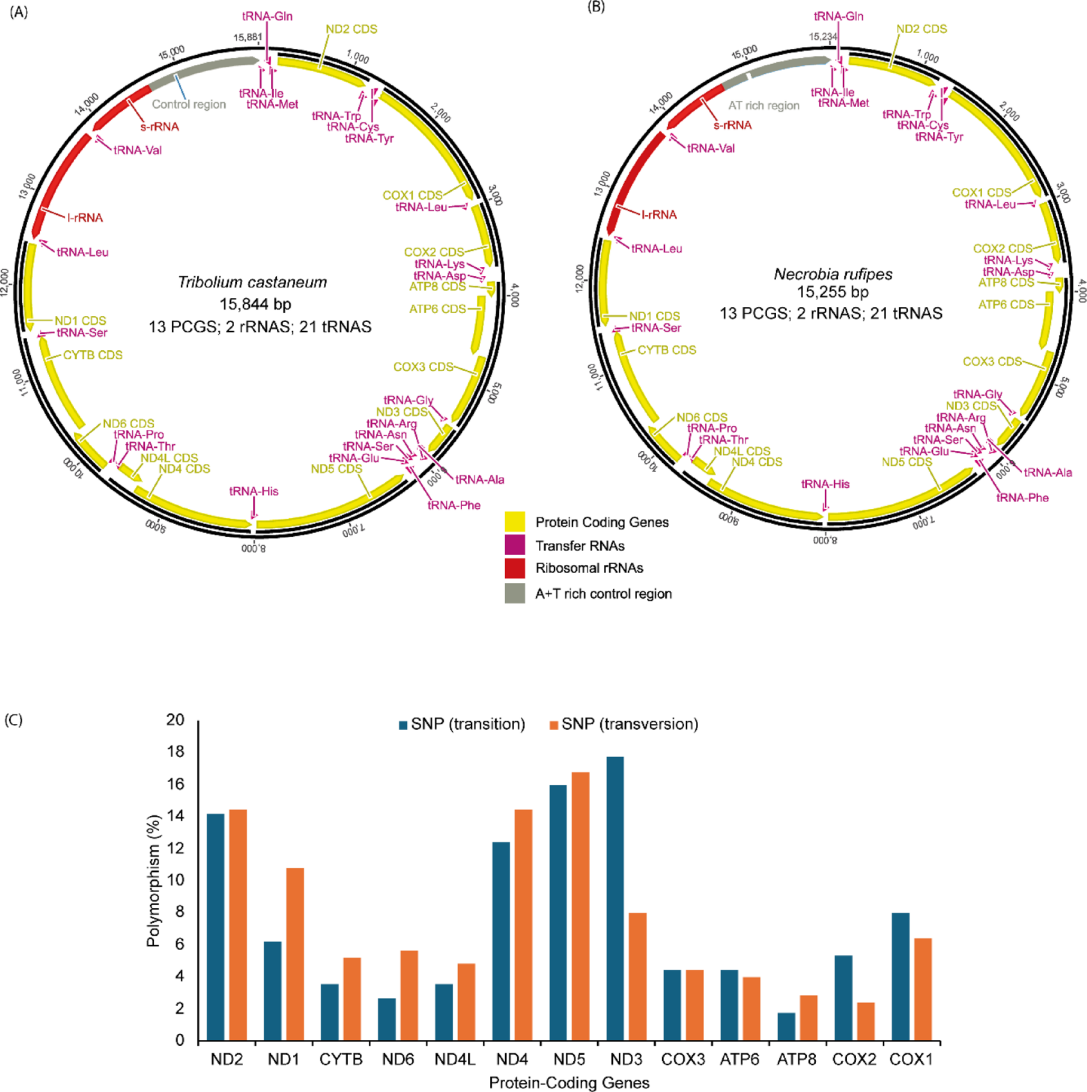
Mitogenome features of *Necrobia rufipes* and *Tribolium castaneum* showing (**a**) circular map of *Tribolium castaneum* mitogenome, (**b**) circular map of *Necrobia rufipes* mitogenome, and (**c**) comparison of the Single Nucleotide Polymorphism (SNP) frequency in the protein-coding genes of *Tribolium castaneum* and *Necrobia rufipes* mitogenomes. Protein-coding genes, transfer RNAs, and ribosomal RNAs are represented with standard abbreviations.

The mitogenomes of both species (*N. rufipes* and *T. castaneum*) were identical in the main features. They had the typical Metazoan complement of 13 protein-coding genes (PCGs), two ribosomal RNA (rRNA) genes, 21 transfer RNA (tRNA) genes, and an AT-rich non-coding region (Fig. 1a,b). The alignment of the complete mitogenomes of *N. rufipes* and *T. castaneum* had 10,493 identical sites with a pairwise identity of 63.7%. Between *N. rufipes* and *T. castaneum*, there were 363 SNPs across the PCGs, which resulted in substitutions. A total of 113 transitions and 250 transversions were observed, with the highest percentage of transitions (14%) observed in ND5 and the highest percentage of transversions (18%) observed in ND3. The lowest percentage of transitions (2%) was observed in ATP8, while the lowest percentage of transversions (2%) was observed in COX2 (Fig. 1c).

The complete sequences for *N. rufipes* had a total length of 15,255 bp. In comparison, *T. castaneum* had a total length of 15,844 bp, with twenty-two genes located on the majority strand (J-strand), and the other 14 genes located on the minority strand (N-strand) in both species (Table 1).

**Table 1:** Mitogenome features of the complete sequence of *Necrobia rufipes* and *Tribolium castaneum*. J—majority strand; N—minority strand. IGS—intergenic regions, with negative values representing overlapping regions.

Region	Code	*Necrobia rufipes*						*Tribolium castaneum*					
		Start	Stop	Coordinates	Length	Strand	IGN	Start	Stop	Coordinates	Length	Strand	IGN
COX1	–	ATT	TAA	1-1560	1560	J	–	AAT	TAA	1-1549	1549	J	− 1
tRNA-Leu	L2			1530-1594	65	J	72			1551-1615	65	J	–
COX2	–	ATC	TAA	1667-2348	682	J	–	ATA	TAA	1616-2299	684	J	–
tRNA-Lys	K			2349-2419	71	J	− 1			2301-2371	71	J	33
tRNA-Asp	D			2419-2485	67	J	− 1			2405-2470	66	J	–
ATP8	–	ATT	TAA	2486-2642	157	J	− 7	ATT	TAG	2471-2626	156	J	− 7
ATP6	–	ATG	TAA	2636-3316	681	J	− 11	ATG	TAA	2620-3291	672	J	− 1
COX3	–	ATT	TAG	3306-4091	786	J	8	ATG	TAA	3291-4076	786	J	2
tRNA-Gly	G			4100-4162	63	J	–			4079-4140	62	J	− 7
ND3	–	ATT	TAA	4163-4517	355	J	− 1	ATA	TAA	4134-4492	359	J	–
tRNA-Ala	A			4517-4582	66	J	− 1			4493-4559	67	J	− 1
tRNA-Arg	R			4582-4646	65	J	− 2			4559-4621	63	J	–
tRNA-Asn	N			4645-4711	67	J	–			4622-4684	63	J	–
tRNA-Ser	S1			4712-4778	67	J	–			4685-4743	59	J	–
tRNA-Glu	E			4779-4840	62	J	–			4727-4793	67	J	− 1
tRNA-Phe	F			4841-4908	68	N	–			4871 − 4808	64	N	–
ND5	–	ATT	T–	4909-6622	1714	N	− 1	ATA	T–	4872-6585	1714	N	− 3
tRNA-His	H			6622-6687	66	N	–			6583-6647	65	N	–
ND4	–	ATA	TAA	6688-8038	1351	N	–	ATG	T–	6648-7980	1333	N	− 7
ND4L	–	ATG	TAA	8039-8323	285	N	− 4	ATG	TAA	7974-8261	288	N	2
tRNA-Thr	T			8328-8392	65	J	–			8264-8326	63	J	2
tRNA-Pro	P			8394-8457	64	N	–			8329-8390	62	N	4
ND6	–	TTA	TAA	8458-8961	504	J	− 1	ATC	TAA	8395-8889	495	J	− 1
CYTB	–	ATG	TAG	8961-10097	1137	J	− 1	ATG	TAA	8889-10026	1138	J	–
tRNA-Ser	S2			10096-10163	68	J	–			10027-10093	67	J	18
ND1	–	ATT	TAA	10164-11132	969	N	–	ATA	TAG	10112-11065	954	N	− 3
tRNA-Leu	L1			11133-11201	69	N	–			11063-11126	64	N	–
l-rRNA	–			11202-12067	866	N	–			11127-12407	1281	N	1
tRNA-Val	V			12068-12134	67	N	–			12409-12477	69	N	1
s-rRNA	–			12135-12913	779	N	–			12479-13252	774	N	–
A-T rich region	–			12914-13822	664	J	–			13253-14491	1239	J	–
tRNA-Ile	I			13823-13884		J	–			14492-14554	63	J	− 3
tRNA-Gln	Q			13885-13953	69	N	–			14552-14620	69	N	–
tRNA-Met	M			13953-14021	69	J	–			14621-14688	68	J	5
ND2	–	ATT	TAA	14022-15032	1011	J	− 1	ATA	TGT	14694-15696	1003	J	–
tRNA-Trp	W			15032-15100	69	J	20			15697-15763	67	J	− 1
tRNA-Cys	C			15121-15189	69	N	3			15763-15823	61	N	2
tRNA-Tyr	Y			15193-15255	65	N	− 2			15826-15884	64	N	− 5

The combined length of the 13 PCGs was 11,140 bp and 11,115 bp for *N. rufipes* and *T. castaneum*, respectively. The longest PCG was ND5 (1714 bp) in both species, while the shortest PCG was ATP8 (*N. rufipes*; 157 bp and *T. castaneum*;156 bp) (Table 1). For both species, the large ribosomal RNA gene (866 bp and 1281 bp for *N. rufipes* and *T. castaneum*, respectively) was located between tRNA^Leu1^ and tRNA^Val^. The small ribosomal RNA gene (779 bp and 774 bp for *N. rufipes* and *T. castaneum*, respectively) was situated between tRNA^Val^ and the AT-rich region. The tRNA sizes varied between 62 bp (tRNA^Glu^) and 71 bp (tRNA^Lys^) for *N. rufipes* and 61 bp (tRNA^Tyr^) and 71 bp (tRNA^Lys^) for *T. castaneum* (Table 1).

*Tribolium castaneum* had thirteen (13) short gene overlaps, mostly involving tRNAs, while *N. rufipes* had twelve (12) gene overlaps. Intergenic regions were found at 10 locations in *T. castaneum*, while intergenic spacers (IGS) were found at five (5) locations in *N. rufipes*. The largest intergenic spacer identified in *T. castaneum* was between tRNA^Lys^ and tRNA^Asp^ (33 bp), while in *N. rufipes*, it was between tRNA^Leu^ and COX2 (72 bp) (Table 1).

### Phylogeny within the family Tenebrionidae

**Fig. 2:**
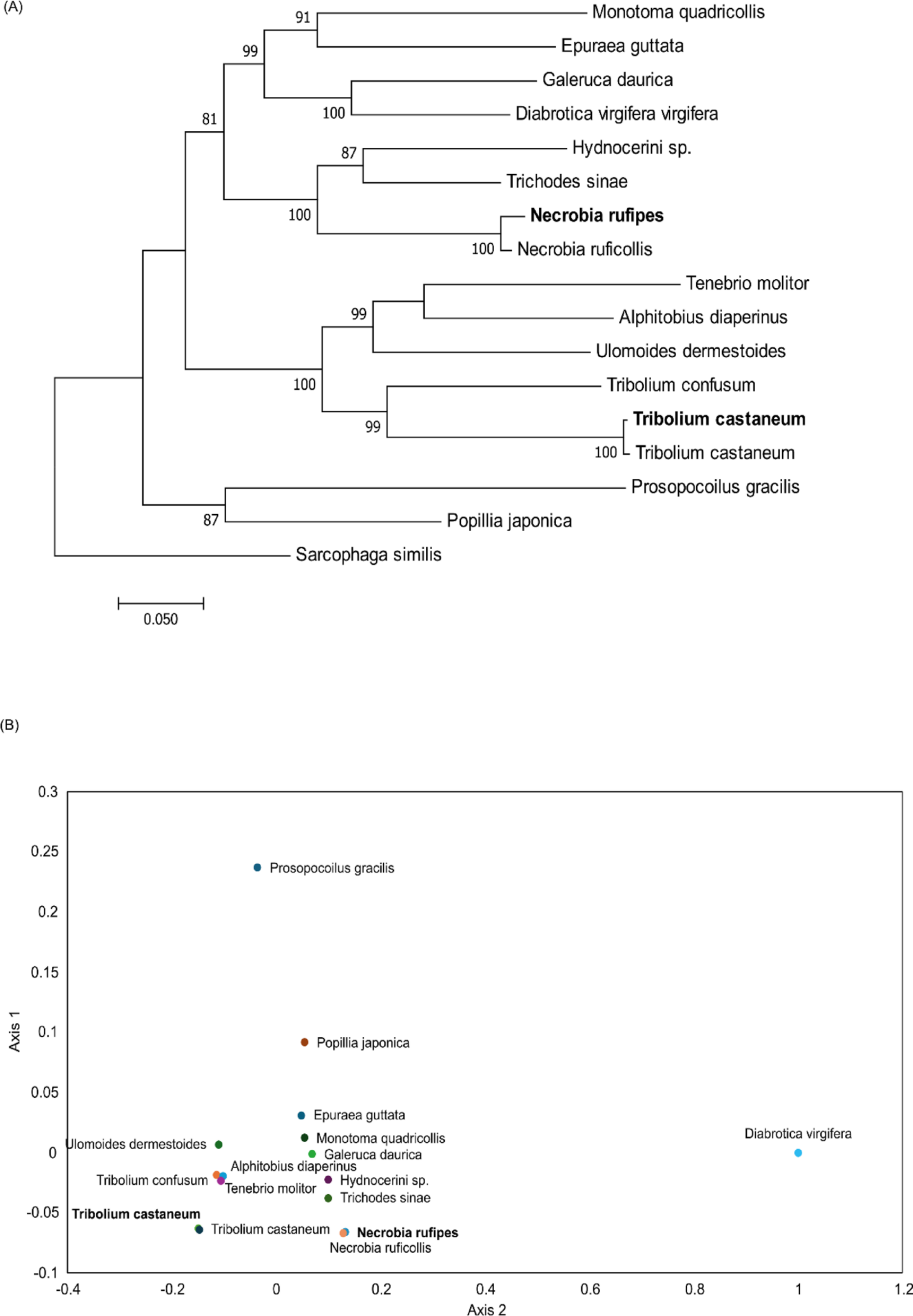
Phylogeny and diversity of *Necrobia rufipes* and *Tribolium castaneum* from this study (in bold) and 14 publicly available Coleopteran sequences using the complete complement of the 13 mitochondrial protein-coding genes. (**A**) The maximum likelihood clustering of *Necrobia rufipes* and *Tribolium castaneum* within the families Cleridae and Tenebrionidae, with *Sarcophaga similis* (Sarcophagidae) as an outgroup, and (**B**) The inter-specific pairwise distances (K2P). Values represent nodal support calculated from 1000 bootstrap replicates.

A maximum likelihood tree was built using the 13 PCGs obtained from the samples in this study combined with PCGs of the representative sequences available in GenBank to assess the phylogenetic structure of the mitogenomes in relation to other Coleopterans (Fig. 2a). The tree topology indicated that the *N. rufipes* and *T. castaneum* mitogenomes were polyphyletic and the *N. rufipes* from this study formed a monophyletic cluster with members of the Cleridae family, *Hydnocerini sp.* and *Trichodes Sinae*, while *T. castaneum* formed a monophyletic cluster with members of the family Tenebrionidae. The interspecific genetic distances between the mitogenomes showed that *T. castaneum* and *N. rufipes* had an average divergence of 32.33% (Fig. 2b).

### Microbiome profiling

**Fig. 3:**
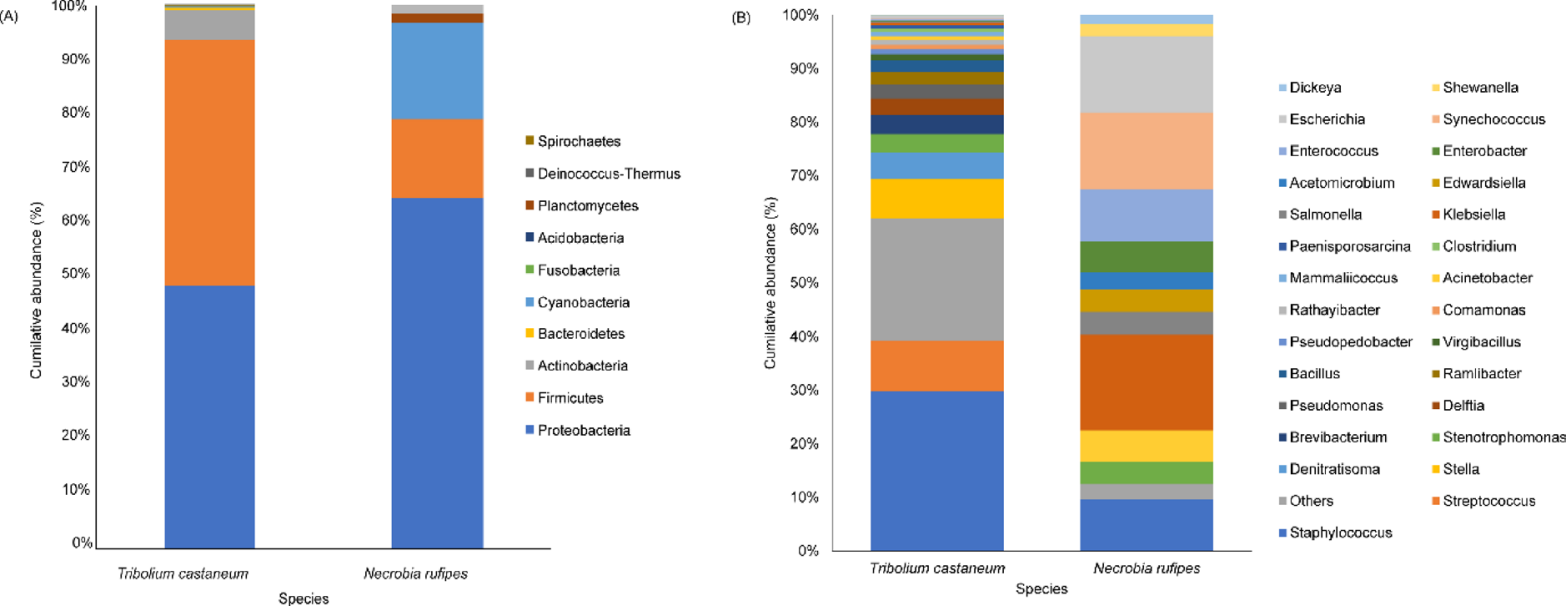
Microbiome diversity statistics showing the relative gut microbial abundance of *Tribolium castaneum* and *Necrobia rufipes* from stored black soldier fly larvae at (**A**) Phylum level and (**B**) Genus level.

The taxonomy and the cumulative abundance of the bacterial genera present in *N. rufipes* and *T. castaneum* showed that the phylum Proteobacteria had the highest abundance, followed by the phylum Firmicutes in both species (Fig. 3a). However, *T. castaneum* had 78% more phyla represented within its microbiome than *N. rufipes*. The most abundant genera in *T. castaneum* were *Staphylococcus* (30.4%), *Streptococcus* (9.6%) and *Stella* (7.5%), while the most abundant genera in *N. rufipes were Klebsiella* (14.2), *Synechococcus* (11.4%), *Escherichia* (11.3%) (Fig. 3b).

### Correlation of most abundant bacterial genera in ***Tribolium castaneum*** and ***Necrobia rufipes***

**Fig. 4:**
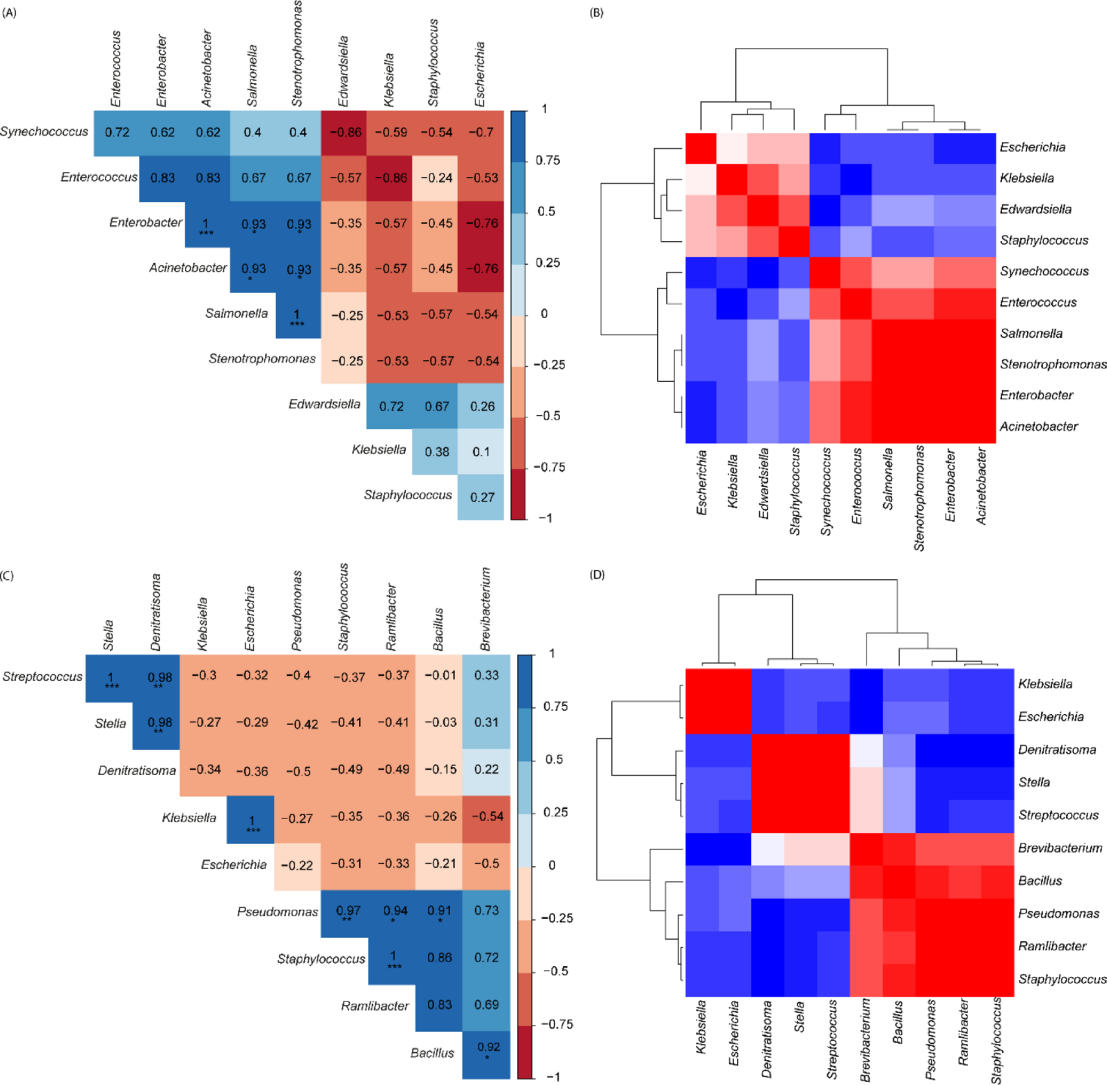
The interplay of the ten most abundant bacterial genera in *Tribolium castaneum* and *Necrobia rufipes*, showing (**A**, **B**) the correlation and heatmap of the most abundant bacterial genera in *Necrobia rufipes*, (**C**, **D**) the correlation and heatmap of the most abundant bacterial genera in *Tribolium castaneum*, obtained from stored black soldier fly larvae.

The analyses of the interplay of the gut microbiota in both species revealed that amongst the most abundant gut bacterial genera in *N. rufipes*, there was a negative correlation (*p* < 0.05) between *Enterococcus*, *Escherichia*, *Staphylococcus* and *Klebsiella*. While a positive correlative relationship was observed between *Synechococcus*/*Enterococcus*, *Escherichia*/*Staphylococcus*, *Escherichia*/*Klebsiella*, and *Staphylococcus*/*Klebsiella* (Fig. 4a,b). Amongst the most abundant gut bacterial genera in *T. castaneum*, *Staphylococcus* had a negative correlation (*p* < 0.05) with the other genera (*Escherichia*, *Klebsiella* and *Stella*) while a strong positive correlation (*p* < 0.05) was observed among the other four genera (Fig. 4c,d).

### Diversity and bacterial community profile of *Tribolium castaneum* and *Necrobia rufipes* microbiomes

**Fig. 5:**
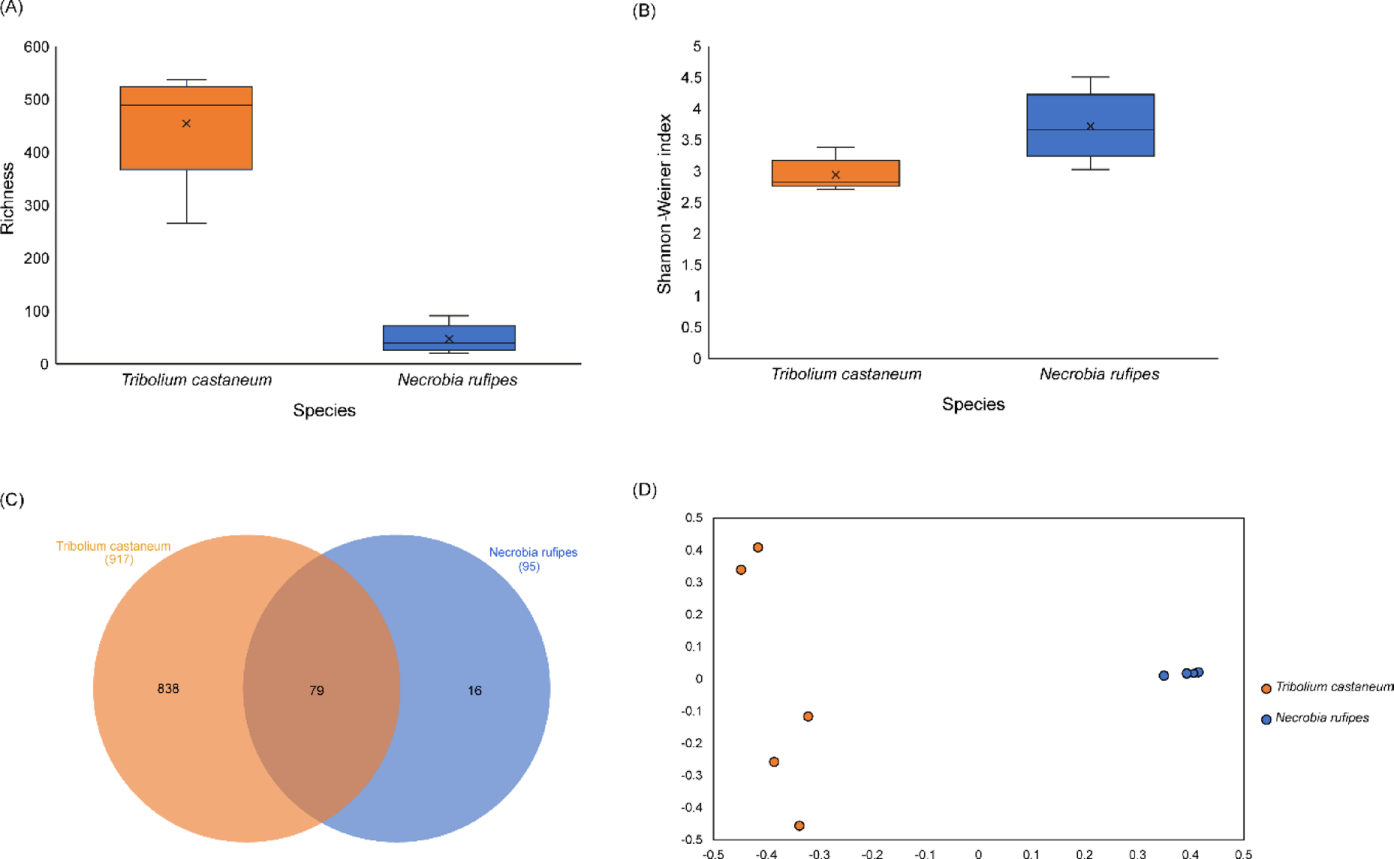
Alpha diversity measures showing (**A**) Richness, (**B**) Shannon diversity index, (**C**) Number of unique and shared bacteria genera within the gut of *Tribolium castaneum* and *Necrobia rufipes* obtained from stored black soldier fly larvae and (**D**) Two-dimensional principal coordinate analyses plot of the beta diversity of bacterial genera in *Tribolium castaneum* and *Necrobia rufipes*, estimated using the Bray-Curtis dissimilarity index.

The analysis of the gut microbial diversity revealed that *T. castaneum* had a higher species richness than *N. rufipes* (Fig. 5a). The Shannon diversity index showed that *N. rufipes* had a higher species diversity than *T. castaneum* (Fig. 5b). A Venn diagram as implemented in InteractiVenn^36^ showed that 79 bacterial genera were common between the two insect groups (Fig. 5c). *Tribolium castaneum* had the most unique number of bacterial genera (838), while the *N. rufipes* had the lowest number of unique genera (16) (Fig. 5c). The beta diversity results showed a close clustering of the bacterial communities of *N. rufipes*, with an average intrapopulation diversity of 0.43, while the bacterial communities of *T. castaneum* were sparsely clustered with an average intrapopulation diversity of 0.99 (Fig. 5d). The two insect species showed high interspecific variance along axis 2 with an average interpopulation distance of 9% (Fig. 5d).

### Correlation between the mitochondrial genome diversity and the microbiome diversity of *Tribolium castaneum* and *Necrobia rufipes*

**Fig. 6:**
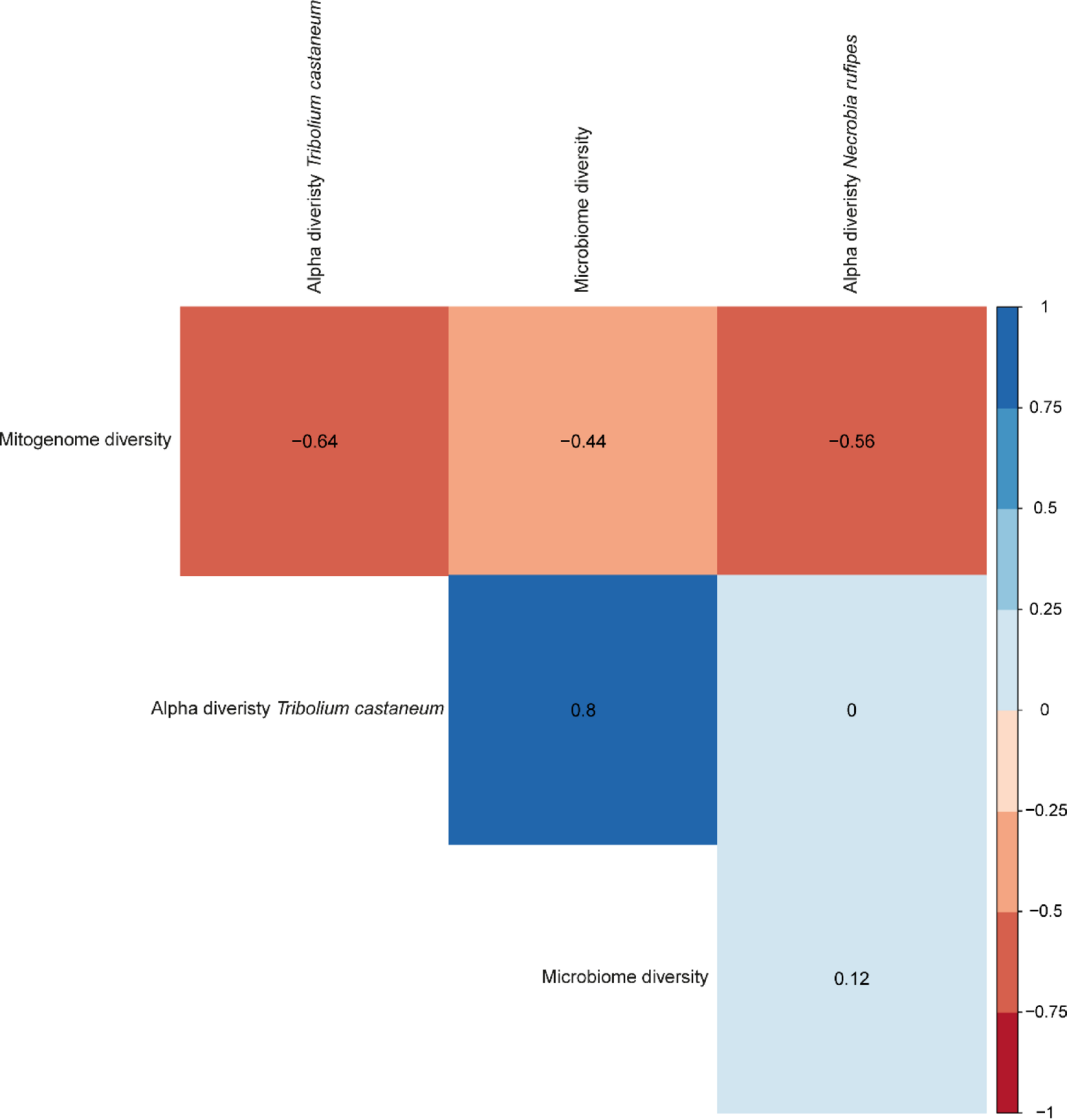
The correlation between the mitogenome diversity and microbiome diversity of *Tribolium castaneum* and *Necrobia rufipes* obtained from stored black soldier fly larvae.

The correlation between genetic diversity and microbiome diversity of *N. rufipes* and *T. castaneum* showed a strong negative correlation (*p* < 0.05) between the genetic diversity and both the alpha and beta diversity of both species (Fig. 6).

## Discussion

Currently, research has focused on the nutritional value and bioconversion capabilities of the BSF. However, BSF-associated pests and pathogens, as well as their potential for transferring contamination in the feed value chain, have been overlooked^5^. To gain insights into the biology of these pests of stored dried BSF larvae products, we assembled the first complete mitochondrial genome sequence from the red-legged ham beetle *N. rufipes* and comparatively evaluated its mitogenome with the mitogenome of the flour beetle *T. castaneum*, which has been used as a model insect for mitogenomic and host-microbiome interaction studies^12,13^. Furthermore, we assessed the microbiome profile of these two storage pests identified on stored BSF larvae and assessed the relationship between the genetic diversity and the gut microbiome diversity of the two species to provide insights into their potential for impeding the safety and quality of stored BSF larvae products.

The *Tribolium* mitogenome sequence has been shown to be a useful resource and entry point for comparative studies of the dynamics of mitochondrial genome evolution within Coleopteran lineages^12^. Therefore, its use in the comparative assessment of the newly reported *N. rufipes* mitogenome provides insights for identifying and developing molecular markers for monitoring both species in stored BSF. Our results show that the *N. rufipes* mitogenome is similar to the *T. castaneum* mitogenome in gene arrangement, and both mitogenomes (*N. rufipes* and *T. castaneum*) are identical in their main features. The complete mitogenome sequence length for *N. rufipes* (15,255 bp) was similar to the average length of other members of the Cleridae family (15,646 bp) but longer than the closely related species *Necrobia ruficolis* (14,443)^27^. In comparison, the *T. castaneum* mitogenome had a total length of 15,844 bp, similar to the average length of other members of the Tenebrionidae family (15,685 bp). A comparative look at the codons between the two species showed 12 PCGs in the *T. castaneum* mitogenome had typical ATN start codons, whereas COX1 had a non-canonical AAT start codon. This was also observed in studies of the *T. castaneum* mitogenomes, as previously reported^12^. Ten out of the 13 PCGs in *N. rufipes* had typical ATN start codons, while ND6 had a TTA start codon and COX1 had an ATT start codon. This has also been observed in the mitogenome of the closely related member of the Cleridae family, *N. ruficolis*^27^. The initiation of COX1 by a different and non-putative start codon has been reported in several insects, including the wild silkworm moth, the seven-spotted lady beetle, the two-spotted stag beetle, and the American black flour beetle^26,31–34^. In our study, the canonical stop codon (TAA or TAG) occurred in 11 PCGs in the *T. castaneum* mitogenome, in agreement with the *T. castaneum* mitogenome reported by Friedrich and Muqim^12^. However, this was in contrast to the *T. castaneum* mitogenome reported by Liu et al.^28^, which had five PCGs terminated by TAA or TAG, while in *N. Rufipes*, the canonical stop codon occurred in 12 PCGs with ND5 terminated by a single T**, which is commonly reported in arthropod mitogenomes^35^. Both mitogenomes were compact; however, one large intergenic spacer in each species’ mitogenome was observed. The longest intergenic spacer found in *N. rufipes*, located between tRNALeu and COX2 (72 bp), was larger than the longest intergenic spacer identified in *T. castaneum* between tRNA^Lys^ and tRNA^Asp^ (33 bp). The presence of large intergenic spacers has been observed in Prosopocoilus stag beetle species (*Prosopocoilus castaneus* (375 bp) and *Prosopocoilus laterotarsus* (158 bp))^36^. Furthermore, although the roles of the IGS coleopterans have not been extensively researched, it is plausible that the presence of IGS in insect mitogenomes may be important for studying species delimitation and evolutionary histories in coleopteran species, including the family Cleridae^36^.

The highly degenerate circular mitochondrial genome found in insects has made molecular markers obtained from mitogenome gene regions widely used in evolutionary studies^23,37^. Our study showed a 36.3% variation between the mitogenomes of *N. rufipes* and *T. castaneum*, with 113 SNPs, and the lowest percentage of SNPs was observed in ATP8. This has also been observed in the mitogenomes of other species, such as *P. castaneus* and *P. laterotarsus*, where ATP8 had the least number of SNPs, which led to amino acid substitutions and the fastest evolutionary rates^36^. Our findings support previous conclusions that rapidly evolving regions of the mitochondrial genome may be informative despite the effect of multiple substitutions^12,36,38^. The conserved regions of the mitochondrial genome have been shown to be informative in resolving taxonomy and ancestry^12,23^. Furthermore, our assessment of the phylogeny of the mitogenomes of the two pest species showed the nodes support a polyphyletic relationship between *N. rufipes* and *T. castaneum*. Furthermore, a monophyletic relationship was seen between the members of the Cleridae family. Likewise, monophyly was observed between members of the Tenebrionidae family. This has also been seen in the assessment of the mitochondrial genomes of members of the Tenebrionidae family and the Tenebrioninae subfamily^12,25,28^. The inclusion of the *N. rufipes* mitogenome from this study expands the genomic database and extends the taxonomic range of available coleopteran mitochondrial genome sequences, thus allowing for deeper resolution of the phylogeny within the order.

A plethora of host-microbial associations exist in insects, whereby insects use symbionts to facilitate numerous physiological and biological functions such as nutrition, provision of metabolic precursors, detoxification, and defense against pathogens and predators^13,39–41^. Our study identified that the most abundant bacterial phylum in both species found on stored BSF larvae was Proteobacteria, followed by Firmicutes. This has also been observed in previous studies on the microbiome of *T. castaneum* and *Cryptorhynchus lapathi*, where Proteobacteria and Firmicutes dominated the microbiome of *T. castaneum* reared on conditioned flour, with Proteobacteria being the most abundant phyla^13,42^. Bacteria within the genera *Staphylococcus* and *Streptococcus* were observed to be the most abundant in *T. castaneum* from our study, while *Klebsiella*,* Synechococcus* and *Escherichia* were observed to be the most abundant in *N. rufipes*. Previous studies have reported *T. castaneum* microbiome composition shift based on diets where bacteria from the genus *Enterococcus* and the Phylum Firmicutes were the most abundant in the beetles fed on flours, and Enterobacteriaceae were abundant in wheat-reared females and females reared in corn^13^. Interestingly, bacteria from the genus Enterococcus were the fifth most abundant in *N. rufipes*, while the genus was observed in low abundance in *T. castaneum* in this study. The bacterial genera *Stenotrophomonas* and *Staphylococcus*, with potential clinical significance, were identified in relatively high abundance in both species (*N. rufipes* and *T. castaneum*). These bacterial genera are rich in siderophores, which are critical metabolites in pathogenic bacteria for iron acquisition^43^. This may provide insights into the flour beetle’s nutritional adaptation to the BSF larvae. Previous studies have implicated *Stenotrophomonas maltophilia* in key roles associated with the fitness of the beetle *Dendroctonus rhizophagus*^44^. Furthermore, the high abundance of *Stenotrophomonas* within the microbiome of *T. castaneum* and *N. Rufipes* needs to be elucidated as this bacterium could be beneficial or pathogenic to these insects. Although our study profiled the microbiome of *T. castaneum* and *N. Rufipes* at the genus level, it does not confirm the presence of pathogenic bacterial species in the pests’ microbiome. However, this study provides a baseline for future studies to examine these pests’ microbiomes at the species level to elucidate their pathogenic transmission potential and implications for food safety. Comparatively, we found that while *T. castaneum* had a richer microbiome, with a higher percentage of unique bacterial genera, *N. rufipes* had a higher species diversity than *T. castaneum*. In addition, while the *N. rufipes* microbiome had minimal variance between samples and the *T. castaneum* microbiome had a moderate to wide variance between samples, it was observed that between the two species, the interspecific microbiome variance was moderate. The higher species diversity observed in the *T. castaneum* microbiome in our study suggests that the pest is more easily colonized by gut microbes^45^. It also implies the pest may be less dependent on host-specialized microbiota than *N. rufipes*. This is consistent with the ecology of *T. castaneum*, as a generalist stored-produce pest with a wide range of substrates, while *N. rufipes* is mainly associated with stored meat products^13,46^. Furthermore, the diversity of microbes in *T. castaneum* may increase the risk of food spoilage and pathogen transmission in stored produce by increasing the likelihood of carrying opportunistic and pathogenic microbes. In addition, a richer microbiome suggests more complex enzymatic interactions in *T. castaneum*, resulting in faster lipid and protein breakdown and the production of volatile organic compounds that signal spoilage^47^.

The intricate associations between the gut microbiome, insect fitness, insect diversification, and evolution have been reported^48^. Also, Studies have shown that variations in the mitochondrial DNA have effects on the gut microbiome, possibly due to differences in reactive oxygen species (ROS) production^49^. Furthermore, genes in the mitochondrial genome coding for adenosine triphosphate (ATP) have been reported to be evolutionarily linked to ROS^50,51^, and nonradical ROS like hydrogen peroxide have been reported to influence gut microbial diversity^52^. However, the correlation between insect mitogenome diversity and gut microbiome diversity is cryptic. Therefore, our study examined the relationship between species diversity and the inter- and intra-population diversity of the microbiomes of both species. Although correlation does not necessarily imply causation, the negative correlation observed between the mitogenome diversity and the bacterial community diversity in this study may suggest that the modulation of the microbiome community of different species found on the same substrate may be related to the genetic variation between the species, as insect genetic diversity has been shown to be influenced by symbiont associations^53,54^. A deeper look into this hypothesis is required, as microbiome diversity can be driven not only by the insect host but also by the environment and diet^55–57^. Furthermore, the microbiome composition and mitochondrial genome in insects have been shown to influence their behaviour and host-range expansion, leading to adaptation to new host species^58^. As seen in phytophagous insects, the gut microbiome influences the exploitation of new hosts^58,59^, and indeed, this could be the case for *N. rufipes* and *T. castaneum* on stored BSF larvae. The mitochondrion has been reported to be intricately associated with numerous metabolic pathways^60–62^. Some of these pathways, including the tricarboxylic acid (TCA) cycle, are involved in carbohydrate, lipid, and protein metabolism. Consequently, the high protein and lipid content in BSF larvae could also be a determining factor for the rapid proliferation of these two storage pests on BSF larvae in storage, as this presents a valuable nutritional source for the pests.

## Conclusions

In summary, this study comparatively assessed the mitochondrial genomes and microbiome profiles of *N. rufipes* and the model insect *T. castaneum*, both of which have been identified as key storage pests of stored dried BSF larvae. The similarity of the mitogenome and microbiome profiles of both pests on stored BSF identified in this study suggests a probable host-adaptation mechanism and the potential of these pests to accelerate spoilage trajectories in stored produce. However, within the scope of our study, a key limitation was the absence of a control group, as the samples were obtained from BSF stores. Therefore, it is difficult to determine whether the observed microbiome composition reflects a specific adaptation to BSF-derived substrates or reflects the inherent microbial profile of these pests regardless of diet. This warrants further studies to provide insights into species-specific interactions and substrate-specific adaptation in the pests. To our knowledge, this study provides the first report of the complete mitochondrial genome of *N. rufipes*. Therefore, this study provides a valuable genetic resource for accurate identification of *N. rufipes* using mitochondrial gene markers. Additionally, the complete mitogenome sequences of these insect pests provide additional information for future studies on Coleopteran infraordinal relationships. The microbiome profiling in our study provides insights into the pests’ microbial communities and a probable link between the pests’ mitochondrial diversity and their gut microbiota. Hence, our study presents a basis for future studies on the functional roles of the gut microbiomes of these insects and information on the potential of insect diversity, in tandem with their microbiomes, to modulate pest behaviour.

## Methods

### Sample collection and DNA extraction

**Fig. 7:**
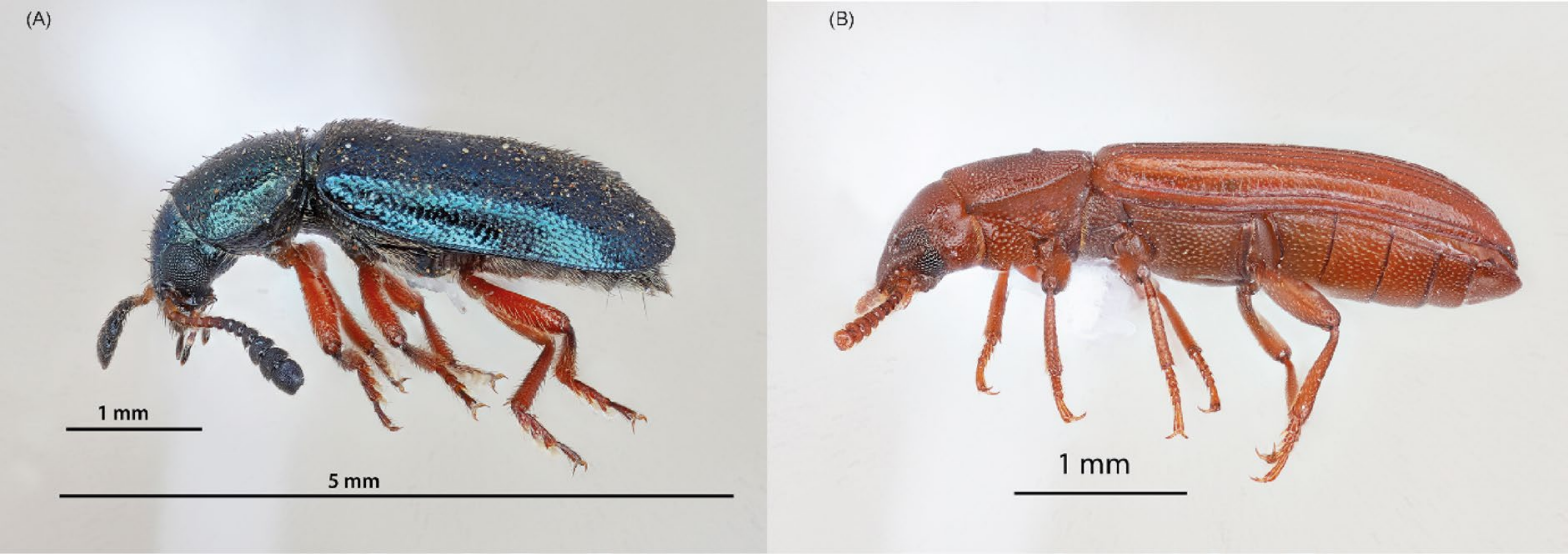
Lateral view of (**A**) adult *Necrobia rufipes* De Geer 1775 and (**B**) adult *Tribolium castaneum* Herbst, 1797.

*Necrobia rufipes* and *Tribolium castaneum* were obtained from stored black soldier fly larvae products and stored in 96% ethanol. One adult specimen representative of *N. rufipes* (Fig. 7a) and *T. castaneum* (Fig. 7b) was photographed, and samples were deposited in the Biosystematics unit of the International Center of Insect Physiology and Ecology (*icipe*). To analyze the mitochondrial genomes of both species, two specimens per species (*N. rufipes* and *T. castaneum*) were randomly selected for next-generation sequencing (NGS). To evaluate the microbiome composition of the pests, six specimens per species were selected for full-length 16 S rRNA metabarcoding. Total DNA was individually extracted from each insect using the Isolate II Genomic DNA Kit (Bioline, London, UK). The extracted DNA were stored at − 20 °C until further analyses.

### Mitogenome sequencing

The complete mitogenome of *N. rufipes* and *T. castaneum* were sequenced, using two specimens per species for NGS. Total DNA from each sample was sequenced separately (whole genome) using the DNBseq sequencing platform at BGI Genomics (BGI, Tai Po, N.T, Hong Kong). De Novo assembly of the *N. rufipes* mitogenome and a reference-based assembly of the *T. castaneum* mitogenome were done using SPAdes v.3.13.0^63^, and the resulting contigs were identified by BLAST + ^64,65^. Subsequently, mapping of the new *T. castaneum* mitogenome was done using the publicly available mitogenome sequence of *T. castaneum* (NC_003081) as a reference in Geneious Prime v2019.1 ^66^ for confirmation. Open reading frames of protein-coding genes (PCGs) were identified in Geneious, using the invertebrate mitochondrial genetic code. Transfer RNAs (tRNAs) were identified using ARWEN software^67^. Intergenic spacers (IGS) and overlapping regions were manually counted.

### Comparison of *Necrobia rufipes* and *Tribolium castaneum* mitogenomes and species diversity

Single-nucleotide polymorphisms (SNPs) within the PCGs among the mitogenomes were assessed using a pairwise sequence alignment using the MAFFT algorithm^68^, of the sequences obtained in this study. Nucleotide pairwise distances (p-distances) were used to calculate the genetic divergence between the sequences in MEGA v. 11.0.9 ^69^ under the Kimura 2-parameter Model^70^.

The phylogeny of the families Cleridae and Tenebrionidae was assessed using the mitogenomes generated in this study and the complete mitogenomes of members of these families available in GenBank, with *Sarcophaga similis* as an outgroup (Table 2). jModelTest2 ^71^ was used to select the evolutionary model, and the Maximum Likelihood (ML) method for phylogenetic reconstruction was implemented in MEGA v. 11.0.9. Nodal support was based on bootstrap analysis with 1000 replicates.

**Table 2:** List of publicly available mitogenome sequences (*n* = 15) used for the phylogeny of the cleridae and Tenebrionidae families. The mitogenome of *Sarcophaga similis* was used as an outgroup.

Species	Family	Genbank accession	Size (bp)
*Trichodes sinae*	Cleridae	NC_033340	16,047
*Necrobia ruficollis*	Cleridae	MK422543	14,443
*Hydnocerini* sp.	Cleridae	KX035157	16,448
*Ulomoides dermestoides*	Tenebrionidae	NC_025332	15,434
*Tribolium confusum*	Tenebrionidae	NC_026702	15,813
*Tribolium castaneum*	Tenebrionidae	NC_003081	15,881
*Tenebrio molitor*	Tenebrionidae	NC_024633	15,785
*Alphitobius diaperinus*	Tenebrionidae	NC_049092	15,511
*Diabrotica virgifera virgifera*	Chrysomeloidae	KF658070	16,650
*Galeruca daurica*	Chrysomeloidae	NC_027114	16,615
*Epuraea guttata*	Nitidulidae	KX087289	16,021
*Monotoma quadricollis*	Monotomidae	NC_036266	16,064
*Prosopocoilus gracilis*	Lucanidae	NC_027580	16,736
*Popillia japonica*	Scarabaeidae	NC_038115	16,541
*Sacrophaga similis*	Sacrophagidae	NC_025573	15,158

Genetic divergences among the species were calculated as pairwise distances (p-distances) in MEGA v. 11.0.9 under the Kimura 2-parameter model (K2P). Inter-specific genetic distances were represented with Multidimensional scaling analysis using the ‘cmdscale’ function in R statistical software version 3.5.1^72^ on the genetic distance matrix to generate the plot for principal coordinate analysis (PCoA).

### Comparison of the gut microbiome of *Necrobia rufipes* and *Tribolium castaneum* adults sample collection

To assess the gut microbiome of *N. rufipes* and *T. castaneum* adults, adult beetles were collected from stored BSF larvae using a manual aspirator preserved in 96% ethanol and kept at − 80 °C awaiting DNA extraction.

### Genomic DNA extraction and 16 S rRNA metabarcoding

Genomic DNA from the insect samples (six samples per species) was extracted using the Isolate II Genomic DNA kit (Bioline, London, U.K). DNA quality and concentration were checked using a Nanodrop 2000/2000c Spectrophotometer (Thermo Fischer Scientific, Wilmington, USA). Sequencing the full-length bacterial 16 S rRNA was performed on an Oxford Nanopore Technology (ONT) MK1C device using 1D^2^ sequencing chemistry (Oxford Nanopore Technologies, Inc., Oxford, UK) on an R9.4 flow cell. DNA Libraries were prepared using the ONT 16 S Barcoding Kit (SQK-16S024) following the manufacturer’s instructions. Libraries were purified and sequencing was performed for four hours. The reads were live base-called using the ONT guppy in the MinKNOW software (v19.05.0). The raw sequencing reads were then uploaded to the ONT cloud for analysis. The FASTQ-WIMP workflow in the EPI2ME software was used to analyse the datasets and assign taxonomies. The cumulative abundance of bacterial phyla and genera was computed and visualised using stacked bar plots. Pearson’s correlation analysis was carried out on the ten most abundant bacterial genera to observe the interplay between abundant bacteria in both species and visualised using correlograms and heatmaps. The alpha diversity metrics, richness and Shannon-Wiener diversity index were computed to evaluate the diversity of the microbiomes within the species, while the beta diversity was calculated using the Bray–Curtis dissimilarity index to evaluate the diversity of the microbiomes between the species. Interpopulation distances were calculated using the Vegan package in R statistical software version 3.5.1.

### Correlation between species diversity and gut microbiome diversity

To determine the relationship between the diversity between the two species and the diversity in their gut microbiome, Pearson’s correlation analysis was performed and a correlation matrix was generated and visualised using a correlogram.

## Data Availability

All sequences generated in this study were deposited in the GenBank database ( [www.ncbi.nlm.nih.gov/genbank](http:/www.ncbi.nlm.nih.gov/genbank) ) under the BioProject number: PRJNA995429 and accession number: OR450807.1.
